# Occupational therapists’ knowledge, attitude, and perceived barriers regarding palliative care: a cross-sectional study in Saudi Arabia

**DOI:** 10.3389/fpubh.2026.1804684

**Published:** 2026-06-17

**Authors:** Amani Alnamnakani, Hassan Izzeddin Sarsak, Olfat Ibrahim Ali, Naif Qasem Aljabri, Sarah Ziyadah, Shouq Almutairi, Tala Almarshoud, Leen Maghrabi, Rahaf Almalky

**Affiliations:** 1 Occupational Therapy Program, Batterjee Medical College, Jeddah, Saudi Arabia; 2 Department of Basic Science for Physical Therapy, Faculty of Physical Therapy, Cairo University, Giza, Egypt; 3 Physical Therapy Program, Batterjee Medical College, Jeddah, Saudi Arabia; 4 Occupational Therapy Department, College of Medical Rehabilitation Sciences, Taibah University, Madinah, Saudi Arabia

**Keywords:** attitude, end of life care, occupational therapy, palliative care, Saudi Arabia

## Abstract

**Background:**

Palliative care (PC) is a specialized healthcare approach dedicated to improving the quality of life for patients and families facing life-threatening illnesses. While global standards emphasize a multidisciplinary team, the specific role of Occupational Therapy (OT) within the Saudi Arabian PC landscape remains under-investigated. This study aims to evaluate the knowledge, attitudes, and perceived barriers of Saudi Occupational therapists (OTs).

**Methodology:**

A cross-sectional online survey was conducted among 50 licensed occupational therapists in Saudi Arabia. Data was analyzed using descriptive statistics and inferential tests, including Pearson’s Chi-square and Independent Samples t-tests, to evaluate the influence of education and experience on PC competencies.

**Results:**

50% of participants demonstrated “average” knowledge, and 80% reported positive attitudes. However, critical gaps were identified, with 94% incorrectly believing PC is only for the terminal stages. Statistical analysis revealed that therapists who received PC training during their university education had significantly higher knowledge scores (*p* = 0.041) than those who did not. The primary barrier to practice was a lack of awareness regarding the OT role (54%).

**Conclusion:**

Although Saudi occupational therapists mainly held favorable views about PC, there were notable knowledge gaps and misunderstandings regarding palliative care. The most significant barrier identified was a lack of awareness of the OT role in a palliative setting. As such, integrating standardized PC modules into OT curricula is vital for raising the awareness of the OT role and aligning with Saudi Arabia’s Vision 2030 healthcare objectives.

## 1 Introduction

Palliative care (PC) is defined by the World Health Organization as an approach that improves the quality of life of patients and their families through the prevention and relief of suffering by means of early identification, impeccable assessment, and treatment of pain and other problems (1). In the context of chronic and life-limiting illnesses, PC is no longer viewed merely as end-of-life care but as a concurrent support system from the time of diagnosis (2–4).

In Saudi Arabia, PC has seen significant progress, evolving from a single unit at King Faisal Specialist Hospital more than 20 years ago to a nationwide network of over 21 palliative units within each Ministry of Health cluster in the country (5). This growth is driven by the Saudi Vision 2030, which seeks to enhance the quality of healthcare and increase life expectancy. Occupational therapists (OTs) are essential members of the PC team (6). They focus on assisting patients to attain their highest level of independence in important occupations (7–10). Their interventions range from supporting occupational participation and management of physical and psychological symptoms to use of adaptive technologies, home modifications and legacy building (10, 11).

For example, a position statement by Occupational Therapy Australia (12) outlined the role of occupational therapy in palliative care. Working within interdisciplinary teams and utilizing core occupational therapy skills, OTs help to optimize the individual’s function, promote dignity and support participation in valued and essential activities of daily living. Consistent with palliative philosophy, OT interventions are developed in collaboration with people with life-limiting conditions and their circles of support to live and die in the place of their choice.

Therefore, integrating PC into OT curricula is necessary, as OTs play a significant role in managing symptoms, enhancing quality of life, and supporting patients and caregivers; however, insufficient educational preparation has been identified as a key barrier to effective practice (10, 13, 14).

Yet, in Saudi Arabia, OTs often face systemic and educational hurdles despite their potential impact. Previous research has suggested that while healthcare providers in the region have positive intentions, their clinical knowledge of PC protocols is often insufficient (15). In addition, the OT programs in Saudi Arabia are relatively recent. Having been introduced only since 2010 and currently offered by just eight universities, consequently, specialized fields such as palliative care remain a significant area of concern in OT curriculum (16). This study is the first to explore the relationship between OT and PC in Saudi Arabia. It aims to bridge the existing gap in the literature by evaluating the knowledge, attitudes, and perceived barriers among Saudi occupational therapists, thereby providing a data-driven foundation for future educational reforms.

In addition, as this study aims to evaluate the knowledge, attitudes, and perceived barriers of Saudi OTs regarding PC, it relied on the Knowledge-Attitude-Practice Model (KAP) to provide a suitable theoretical framework. It explains how OTs’ knowledge shapes their attitudes, which in turn influences their professional practices (17). In the context of PC, OTs’ level of knowledge about palliative principles is likely to shape their attitudes toward its significance and their role within this practice. These attitudes consequently influence their willingness and ability to engage in PC provision. Furthermore, perceived barriers can be understood within this framework as factors that may hinder the translation of knowledge and positive attitudes into clinical practice. Therefore, the KAP model provides a clear and structured approach to evaluate the relationships between knowledge, attitudes, perceived barriers, and misconceptions while also supporting the alignment of these constructs with the measurement instrument used in this study.

## 2 Materials and methods

### 2.1 Study design and participants

A cross-sectional descriptive design was employed. A total of 50 licensed OTs currently practicing in Saudi Arabia participated. The data collection was conducted from January 2025 to May 2025. The study used a combination of convenience and snowball sampling to ensure maximum reach of the target professional population. Convenience sampling was primarily utilized by distributing the survey across professional platforms (e.g., WhatsApp, LinkedIn, Telegram, and Email) to recruit the participants who met the inclusion criteria. To increase the sample size and reach more respondents, snowball sampling was then employed; primary respondents were encouraged to forward the survey link to their peers within their professional networks. While we acknowledge the non-probability nature of this approach, it was considered the most effective method for accessing a specific sample of OT specialists.

### 2.2 Instrumentation

The study utilized a 32-item questionnaire adapted from Chew et al. (7), which included four sections as follows: Section 1: Demographics (8 items), including age, gender, education, and years of experience; Section 2: Knowledge (12 items), presented in a True/False/Do not Know format. Scoring: 0–4 (Low), 5–8 (Average), 9–12 (High). The questions assessed participants’ comprehension of fundamental concepts related to palliative care, including its definition, purpose, scope of services, patient needs, and prevalent misconceptions. Each correct response was awarded a score of one, while incorrect responses were assigned a score of zero, resulting in a total possible score ranging from 0 to 12. Higher scores indicated a more comprehensive understanding of palliative care. Misconceptions were identified based on frequently incorrect responses to specific items.

Section 3: Attitude (13 items): 5-point Likert scale (1 = Strongly Disagree to 5 = Strongly Agree). The attitude section comprised 13 statements aimed at evaluating participants’ perceptions, feelings, and readiness regarding the provision of palliative care. These items assessed various dimensions of attitude, including comfort in interacting with patients (Q1, Q2, Q6), perceived knowledge and understanding (Q3, Q13), perceived value and beliefs about palliative care (Q4, Q7, Q9), emotional responses (Q8), perceived difficulty (Q5), communication about end-of-life issues (Q10), and self-perceived emotional and clinical preparedness (Q11, Q12). Participants responded using a five-point Likert scale ranging from strongly disagree to strongly agree.

Section 4: Barriers (Multiple Choice): Identified obstacles preventing PC involvement; Participants were asked to identify the primary barrier impeding the practice of occupational therapy within palliative care settings. The response options provided included: a lack of awareness regarding the necessity of occupational therapy in palliative contexts, a lack of interest among occupational therapists in working within such settings, confusion concerning the role of occupational therapy in palliative care, insufficient reimbursement, and an “others” category for additional responses. Each participant selected one response, and the data were subsequently analyzed using frequency and percentage distribution (Appendix).

The validity and reliability of the questionnaire were adopted from a previously developed questionnaire (9) as it has a good reliability (internal consistency value is at 0.88), and the contents has been validated by experts in palliative care (9).

The analysis of the questionnaire identified the misconceptions as erroneous beliefs concerning palliative care, as evidenced by inaccurate responses in the knowledge section and negatively framed or misleading perceptions in the attitude section. Common misconceptions included the belief that palliative care is exclusively for terminally ill patients, that referral to palliative care indicates the cessation of treatment, and that palliative care is intended for individuals without hope. Furthermore, confusion regarding the role of occupational therapy in palliative care was identified as a significant misconception among respondents.

### 2.3 Statistical analysis

Data were analyzed using SPSS version 25. Descriptive statistics (means, standard deviations, and percentages) summarized the primary findings. Chi-square test (𝜒2) used to determine if there was a significant association between “PC training in University” (Yes/No) and “Knowledge Level” (Low/Average/High). An independent t-test was conducted to compare the mean attitude scores between male and female therapists to check for gender-based differences in perspectives.

### 2.4 Ethical considerations

This study was approved by the Research Ethics Committee at Batterjee Medical College in Saudi Arabia (RES-2023-0088). Participation in the study was fully voluntary, and therapists had the freedom to decide whether to take part or not. All responses were kept confidential and anonymous. Only the survey responses were reviewed. Informed consent for the study was obtained electronically, where participants must click “I agree” before continuing. The contact details for the research team were also provided.

## 3 Results

### 3.1 Demographic profile

The participants were predominantly male (62%) and young, with 80% falling in the 20–30 age bracket. While 62% held a bachelor’s degree, 60% admitted to having “rare” or “no” experience with PC patients in their daily practice (see Table 1).

**Table 1:** Demographic characteristics of respondents (*N* = 50).

Characteristics	Number	Percentage (%)
Gender		
Male	31	62%
Female	19	38%
Age		
20–30	40	80%
31–40	8	16%
41 and above	2	4%
Highest academic qualification		
Diploma	2	4%
Bachelor’s degree	31	62%
Master	13	26%
Doctorate	4	8%
Experience with a palliative patient		
Never	12	24%
Rarely	30	60%
Frequently	6	12%
All the time	2	4%
Training in palliative care		
Never	16	32%
University curriculum	26	52%
Conference	1	2%
Specialized training	7	14%
Employment history		
<1 year	9	18%
1–3 years	20	40%
4–6 years	9	18%
More than 6 years	12	24%

### 3.2 Knowledge and misconceptions

Only 46% of the sample achieved “High” knowledge scores. A major knowledge gap was identified: 94% of respondents incorrectly believed PC should only be provided when a patient is terminal. Furthermore, 66% mistakenly believed that choosing end-of-life care implies the patient has “given up” on life (see Table 2).

**Table 2:** Summary of responses to the palliative care knowledge questionnaire.

Questions	True Number (%)	False Number (%)
Q1: I interpret palliative care as a type of medical treatment offered to someone who has a terminal illness.	47 (94%)	3 (6%)
Q2: I perceive palliative care as a means of alleviating pain for someone who has a terminal condition.	45 (90%)	5 (10%)
Q3: I interpret palliative care as either home-based assistance or care provided in a nursing facility.	38 (76%)	12 (24%)
Q4: I interpret palliative care as providing assistance and guidance for individuals nearing their death.	38 (76%)	12 (24%)
Q5: Each individual who is receiving palliative care has unique requirements.	44 (88%)	6 (12%)
Q6: Understanding the background of individuals who receive palliative care is crucial.	50 (100%)	0 (0%)
Q7: Individuals who are in palliative care can find joy in life.	46 (92%)	4 (8%)
Q8: There are many ways we can enhance the quality of life for individuals receiving palliative care.	48 (96%)	2 (4%)
Q9: Selecting end-of-life care indicates that the patient has come to terms with their impending death.	33 (66%)	17 (34%)
Q10: Referring a patient to palliative care signifies that the physician has “abandoned” the effort to deliver treatment.	15 (30%)	35 (70%)
Q11: End-of-life refers to individuals who have just a few days remaining in their lives.	16 (32%)	34 (68%)
Q12: Palliative care is designed for patients who do not require intricate medical treatment.	23 (46%)	27 (54%)

### 3.3 Attitudes

80% of OTs held positive attitudes toward the necessity of PC. However, 66% felt uncomfortable discussing death with a patient. The descriptive analysis of the item scale reveals a significant discrepancy between the participants’ strong ethical commitment and their clinical or emotional readiness. While there was a near-unanimous consensus on patient rights (82% agreement) and a majority 60% found the work rewarding, participants stated a lack of confidence in communication, with 66% reporting that they do not find it easy to discuss death with patients. Furthermore, although 56% claimed a strong general understanding of palliative care (Q3), only 44% could confidently recognize it from end-of-life care, and 44% still wrongly associated the field with situations where there is “no hope.” This gap is further proved by the fact that only 50% felt clinically or emotionally prepared for such care (Table 3).

**Table 3:** Attitudes of occupational therapists toward palliative care.

Questions	Strongly agree	Agree	Neutral	Disagree	Strongly disagree	Mean score (SD)
Q1: I feel assured when I am around individuals who are in palliative care.	8 (16%)	12 (24%)	25 (50%)	4 (8%)	1 (2%)	3.44 (0.92)
Q2: I feel at ease when physically interacting with individuals in palliative care.	9 (18%)	12 (24%)	18 (36%)	10 (20%)	1 (2%)	3.36 (1.05)
Q3: I have a strong understanding of palliative care.	8 (16%)	20 (40%)	13 (26%)	7 (14%)	2 (4%)	3.50 (1.04)
Q4: It is rewarding to interact with people who are receiving palliative care.	11 (22%)	19 (38%)	14 (28%)	5 (10%)	1 (2%)	3.68 (0.98)
Q5: I find it difficult to imagine caring for someone in palliative care.	4 (8%)	18 (36%)	11 (22%)	10 (20%)	7 (14%)	3.04 (1.16)
Q6: It can be fun to interact with people receiving palliative care and their sentimental belongings.	7 (14%)	14 (28%)	14 (28%)	12 (24%)	3 (6%)	3.20 (1.10)
Q7: Clients receiving palliative care have the right to the same level of care as any other patient.	32 (64%)	9 (18%)	6 (12%)	2 (4%)	1 (2%)	4.38 (0.89)
Q8: Caring for terminally ill patients can be heartbreaking and disheartening.	12 (24%)	25 (50%)	10 (20%)	2 (4%)	1 (2%)	3.90 (0.91)
Q9: Palliative care is intended for individuals who are in situations where there is “no hope.”	2 (4%)	20 (40%)	12 (24%)	10 (20%)	6 (12%)	3.04 (1.10)
Q10: I find it easy to discuss death with a patient who is nearing the end of life.	1 (2%)	7 (14%)	9 (18%)	18 (36%)	15 (30%)	2.22 (1.20)
Q11: I believe I am emotionally ready to provide care for a patient who is nearing the end of life.	10 (20%)	15 (30%)	8 (16%)	12 (24%)	5 (10%)	3.26 (1.14)
Q12: I believe I am ready clinically to provide care for patients who are nearing the end of life.	7 (14%)	18 (36%)	15 (30%)	6 (12%)	4 (18%)	3.36 (1.06)
Q13: I grasp the difference between palliative care and end-of-life care.	8 (16%)	14 (28%)	14 (28%)	11 (22%)	3 (6%)	3.26 (1.08)

### 3.4 Barriers

The primary barrier to OT involvement was the lack of awareness of the OT role (54%), followed by a lack of specialized training (28%). Among all respondents, 16% reported being involved in palliative care “frequently” or “all the time.” Additionally, 54% of participants agreed that the primary barrier preventing occupational therapists from expanding their role in palliative care is the low awareness of the need for occupational therapy in this setting (see Table 4). Furthermore, 28% expressed confusion about the role of occupational therapists in palliative care. On the other hand, 12% indicated a lack of interest in pursuing work in this area. Some participants also added that there was a lack of service reimbursement (see Table 4).

**Table 4:** Barriers to occupational therapy in palliative care.

Variables	Number	Percentage (%)
Lack of awareness of the need for occupational therapy in a palliative setting	27	54%
Lack of interest from OT to work in a palliative setting	6	12%
Confusion of role in a palliative setting	14	28%
Lack of reimbursement	3	6%
Others	0	0%

### 3.5 Statistical inference

The study found a statistically significant relationship between educational background and PC knowledge. Therapists who received PC education at the university level were significantly more likely to score in the “High” knowledge category compared to those with no formal training (𝜒2 = 6.42, df = 2, *p* = 0.041). However, an independent t-test showed no significant difference in attitude and barrier scores between genders (*p* > 0.05), indicating a broadly positive professional outlook across the board. Furthermore, no significant difference in attitude toward PC between males and females (*p* < 0.05).

## 4 Discussion

The findings of this study provide a critical snapshot of the current state of Palliative Care (PC) within the Occupational Therapy (OT) profession in Saudi Arabia. While the transition toward modern PC services is well underway under the Saudi Vision 2030 healthcare transformation, the results reveal a significant disconnect between professional willingness and clinical competence.

### 4.1 The knowledge-practice gap, attitude and the “terminal care” myth

Following the KAP model, knowledge is expected to shape attitude. However, the findings of this study indicate a partial disconnect between these domains. Despite a majority of OTs expressing positive attitudes toward the importance of palliative care (80%), substantial knowledge gaps and misconceptions were evident. For example, striking 94% of participants incorrectly identified PC as a service reserved exclusively for terminal stages. This misconception is significantly higher than the 34.6% reported in similar studies in Malaysia (7), suggesting that in Saudi Arabia, PC is still culturally conflated with hospice or “end-of-life” care. This is a critical barrier, as modern PC frameworks advocate for early intervention at the point of diagnosis to manage chronic symptoms and improve long-term outcomes (WHO, 2020). In addition, this misconception seems to influence attitudes at a deeper level, especially in areas requiring emotional and clinical engagement, as reflected by the high proportion of OTs who felt uncomfortable discussing death (66%) and lacked confidence in communication.

This suggests that while general attitudes toward PC are favorable, inaccurate or insufficient knowledge may compromise OTs’ confidence and emotional readiness. As such, limiting the full development of positive, rational, and practice-oriented attitudes as proposed by the KAP model.

Furthermore, 66% of respondents believed that choosing PC implies a patient has “given up.” This reflects a lack of understanding regarding the “person-centred approach” defined in recent global policies, which views PC as a layer of support that empowers patient autonomy rather than signifying medical failure (3).

### 4.2 The impact of academic training on palliative care

The Chi-square analysis (𝜒2 = 6.42, *p* = 0.041) conducted in this study provides empirical evidence that university-level exposure is the strongest predictor of PC knowledge. Those who received formal training scored significantly higher, yet even within this group, “average” knowledge was common. This suggests a gap in translating knowledge and attitudes into practice. In other words, while PC is being introduced in Saudi OT programs, the depth of the curriculum may be insufficient. Education must move beyond the definition of PC and into the clinical applications of OT, such as energy conservation, home modification for the frail, and spiritual coping mechanisms (11).

### 4.3 Emotional readiness and cultural barriers

Despite a 50% readiness rate in terms of attitude, 66% of OTs reported significant discomfort in discussing death. This “death anxiety” is common among young practitioners (80% of our sample were aged 20–30) and is often rooted in a lack of training in communication protocols. Navigating the “acceptance of death” is a complex clinical process that requires more than just empathy; it requires structured communication skills to help patients find meaning in their final stages (18–20).

Culturally, in Saudi Arabia, discussing death can sometimes be viewed as removing hope, which explains why 40% of our participants were neutral or agreed that PC is for those with “no hope.” These findings align with Ahmed et al. (15), who noted that cultural sensitivities in the Middle East often dictate how palliative information is shared between clinicians and families.

### 4.4 Role confusion and systematic barriers

The most significant barrier identified was a lack of awareness of the OT role (54%). In many Saudi PC units, the multidisciplinary team is often dominated by physicians and nurses, with OTs underutilized or relegated to basic physical rehabilitation. This role confusion stems from a lack of clear clinical pathways and institutional policies that define OT’s contribution to symptom management and quality-of-life enhancement. Without professional advocacy and clearer role definition within the Ministry of Health, OTs will continue to face barriers in accessing the patients who need them most (5, 21).

### 4.5 Comparison with international data

Interestingly, Saudi OTs showed a much more positive attitude toward physical interaction and emotional support compared to their Malaysian counterparts (42% vs. 5.5% in certain attitude metrics). This suggests a high level of professional “buy-in” and compassion within the Saudi OT workforce. The challenge, therefore, is not changing the hearts of the therapists, but rather equipping their heads with the technical knowledge and their hands with the clinical tools necessary to practice effectively in a PC setting.

### 4.6 Limitations

This research has certain limitations: a small sample size, a localized geographic scope, and a lack of existing studies in Saudi Arabia. Although the number of research articles on palliative care in Saudi Arabia is gradually increasing, a shortage of comprehensive studies and extensive national statistics remains. These challenges hindered our attempts to locate relevant literature and contextual frameworks, which may have influenced the depth and general significance of our findings. Additionally, the applicability of our findings is limited by the reliance on convenience and snowball sampling methods, which led to a participant group predominantly composed of digitally engaged individuals between the ages of 20 and 30. Future studies should utilize stratified random sampling to achieve a more equitable representation of all age groups.

### 4.7 Clinical implications and recommendations for practice, education, and future research

#### 4.7.1 Policy innovation and clinical contributions: bridging the role-confusion gap

The study highlights that while OTs in Saudi Arabia possess a high degree of empathy and positive intent, they are significantly underutilized in PC settings. Therefore, there is a need for policy innovation to address the underutilization and role ambiguity of OTs in PC in Saudi Arabia as per the following:

- Standardization of OT roles: There is an urgent need to define the specific clinical contributions of OTs in multidisciplinary PC teams. This includes non-pharmacological pain management, energy conservation for cancer-related fatigue, and home environment modification to sustain independence (11).
- Early intervention advocacy: Clinical pathways must be redesigned to integrate OT at the point of diagnosis rather than during the terminal phase. This shifts the perception of OT from “end-of-life support” to “quality-of-life enhancement” (WHO, 2020).
- Communication skills: Given that 66% of OTs felt unready to discuss death, clinical settings should adopt structured communication frameworks (e.g., the SPIKES protocol) to help OTs navigate difficult conversations with patients and families (19).

These changes are necessary to translate positive attitudes into effective and optimal clinical practice.

#### 4.7.2 Theoretical and educational implications: recommendations for academic intervention

The statistical significance (*p* < 0.05) found between university training and knowledge levels confirms that the academic environment is the primary driver of competency.

Curriculum Reform: Higher education institutions in Saudi Arabia should integrate mandatory Palliative Care modules into the Bachelor of Occupational Therapy (BOT) curriculum. This should move beyond theoretical definitions to include case-based learning on symptom management.

Continuing Professional Development (CPD): For the existing workforce (80% of whom are young professionals), the Ministry of Health and professional associations should provide specialized workshops focusing on “Death Anxiety” and psychosocial coping strategies.

Interprofessional Education (IPE): Training should be conducted alongside nurses and physicians to reduce the “role confusion” reported by 54% of respondents, fostering a team-based understanding of each professional’s scope.

#### 4.7.3 Recommendations for future research

- Qualitative exploration: Future studies should use semi-structured interviews to explore the deep-seated cultural reasons behind why 94% of OTs associate PC only with “no hope” or terminality.
- Longitudinal assessment: Research should track the impact of new PC educational interventions on the clinical confidence of OT graduates over a 2–3-year period.
- Patient-centered studies: It is recommended to investigate the perceptions of patients and families regarding the impact of OT interventions on their satisfaction with PC services in Saudi hospitals.

#### 4.7.4 Framework for practical integration of palliative care

See Table 5 for a summary of strategic recommendations for OT in palliative care. To visualize the strategic actions outlined in the table, here is a breakdown of the recommendations designed to transform the role of Occupational Therapy (OT) in Saudi Arabian palliative care (PC):

**Table 5:** Summary of final strategic actions and recommendations for OT in palliative care.

Category	Strategic action	Objective	Targeted change
Policy	Establish occupational therapy as a core member of the palliative care multidisciplinary team within Ministry of Health (MOH) protocols.	To integrate occupational therapy into MOH palliative care multidisciplinary protocols.	Mandate inclusion
Academic	Standardize palliative care modules across all Saudi occupational therapy bachelor programs to ensure foundational competency.	Move from theoretical definitions to case-based symptom management.	Curriculum reform
Clinical	Implement the SPIKES protocol for structured end-of-life communication training to address the 66% of OTs feeling unready to discuss death.	Shift the perception of OT from “end-of-life” to “quality-of-life enhancement.”	Early intervention
Cultural	Launch awareness campaigns specifically designed to decouple “Palliative Care” from the concept of “Giving up hope.”	Investigate deep-seated cultural reasons behind terminality misconceptions.	Qualitative exploration

Table 5 categorizes the necessary interventions to move the profession from “willing observers” to “competent team members” with strategic action plan that incorporates (1) Policy Initiatives: The Ministry of Health (MOH) protocols should formally establish OTs as core members of the multidisciplinary PC team, (2) Academic Reform: Educational institutions must standardize mandatory PC modules within all Saudi OT bachelor’s programs, (3) Clinical Training: To address the 66% of OTs who feel unready to discuss death, clinical settings should implement the *SPIKES protocol* for end-of-life communication training, and (4) Cultural Awareness: Launching targeted campaigns is essential to decouple the term “Palliative Care” from the misconception of “giving up hope.” Implementing a roadmap based on the statistical findings, the academic sector is the primary key driver for change, as therapists with university training scored significantly higher in knowledge levels (*p* = 0.041).

## 5 Conclusion

In Saudi Arabia, the role of OT within the PC landscape remains under-investigated. This study aims to evaluate the knowledge, attitudes, and perceived barriers of Saudi OTs. The result showed that the “average” knowledge level and the high rate of role confusion are the primary inhibitors to effective OT practice in Saudi PC. The significant correlation between university training and knowledge scores indicates that the solution lies in the academic sector. By standardizing PC education and introducing communication-based workshops, Saudi Arabia can transform its OT workforce from willing observers into vital, competent members of the palliative multidisciplinary team in line with healthcare goals of Vision 2030.

## Data Availability

The raw data supporting the conclusions of this article will be made available by the authors, without undue reservation.
