# Supplementary material for: Occupational therapists’ knowledge, attitude, and perceived barriers regarding palliative care: a cross-sectional study in Saudi Arabia
Source: Front Public Health. 2026 Jun 17;14:1804684. doi: 10.3389/fpubh.2026.1804684 (PMC13318899; doi:10.3389/fpubh.2026.1804684)
Supplement: Supplementary file 1 [file Supplementary_file_1.pdf]

Appendix

Section 1: Demographic section

Age

- 20-30
- 31-40
- 41 or above

Gender

- Male
- Female

Highest academic Qualification

- Diploma
- Degree
- Master
- Doctorate

Employment History

- Less than 1 year
- 1-3 years
- 4-6 years
- More than 6 years

Experience with palliative patients

- Never
- Rarely
- Frequently
- All the time

Training in palliative care

- Never
- University curriculum
- Conference
- Specialized Training

Section 2: Palliative Care Knowledge Questionnaire Section

I interpret palliative care as a type of medical treatment offered to someone who has a terminal illness.

- True
- False

I perceive palliative care as a means of alleviating pain for someone who has a terminal condition.

- True
- False

I interpret palliative care as either home-based assistance or care provided in a nursing facility.

- True
- False

I interpret palliative care as providing assistance and guidance for individuals nearing their death.

- True
- False

Each individual who is receiving palliative care has unique requirements.

- True
- False

Understanding the background of individuals who receive palliative care is crucial.

- True
- False

Individuals who are in palliative care can find joy in life.

- True
- False

There are many ways we can enhance the quality of life for individuals receiving palliative care.

- True
- False

Selecting end-of-life care indicates that the patient has come to terms with their impending death.

- True
- False

Referring a patient to palliative care signifies that the physician has "abandoned" the effort to deliver treatment.

- True
- False

End-of-life refers to individuals who have just a few days remaining in their lives.

- True
- False

Palliative care is designed for patients who do not require intricate medical treatment.

- True
- False

<u>Section 3: Attitude towards Palliative Care Questionnaire Section</u>

I feel assured when I am around individuals who are in palliative care.

- Strongly Agree
- Agree
- Neutral
- Disagree
- Strongly Disagree

I feel at ease when physically interacting with individuals in palliative care.

- Strongly Agree
- Agree
- Neutral
- Disagree
- Strongly Disagree

I have a strong understanding of palliative care.

- Strongly Agree
- Agree
- Neutral
- Disagree
- Strongly Disagree

It is rewarding to interact with people who are receiving palliative care.

- Strongly Agree
- Agree
- Neutral
- Disagree
- Strongly Disagree

I find it difficult to imagine caring for someone in palliative care.

- Strongly Agree
- Agree
- Neutral
- Disagree
- Strongly Disagree

It can be fun to interact with people receiving palliative care and their sentimental belongings.

- Strongly Agree
- Agree
- Neutral
- Disagree
- Strongly Disagree

Clients receiving palliative care have the right to the same level of care as any other patient.

- Strongly Agree
- Agree

- Neutral
- Disagree
- Strongly Disagree

Caring for terminally ill patients can be heartbreaking and disheartening.

- Strongly Agree
- Agree
- Neutral
- Disagree
- Strongly Disagree

Palliative care is intended for individuals who are in situations where there is "no hope."

- Strongly Agree
- Agree
- Neutral
- Disagree
- Strongly Disagree

I find it easy to discuss death with a patient who is nearing the end of life.

- Strongly Agree
- Agree
- Neutral
- Disagree
- Strongly Disagree

I believe I am emotionally ready to provide care for a patient who is nearing the end of life.

- Strongly Agree
- Agree
- Neutral
- Disagree
- Strongly Disagree

I believe I am ready clinically to provide care for patients who are nearing the end of life.

- Strongly Agree
- Agree
- Neutral
- Disagree
- Strongly Disagree

I grasp the difference between palliative care and end-of-life care.

- Strongly Agree
- Agree
- Neutral
- Disagree
- Strongly Disagree

<u>Section 4: Perceived Barriers to Palliative Care Questionnaire Section</u>

In your opinion, what are the variables that create barriers for occupational therapists to participate in palliative care?

- Lack of awareness of a need of occupational therapy in palliative setting
- Lack of interest from OT to work in palliative setting
- Confusion of role in palliative setting
- Lack of reimbursement
- Others
